# Supplementary material for: High-order harmonics measured by the photon statistics of the infrared driving-field exiting the atomic medium
Source: Nat Commun. 2017 Apr 27;8:15170. doi: 10.1038/ncomms15170 (PMC5414173; doi:10.1038/ncomms15170)
Supplement: Supplementary Information — Supplementary Figures, Supplementary Notes and Supplementary References. [file ncomms15170-s1.pdf]

Supplementary Note 1 | On the full quantum optical approach of HOH generation process

The full quantum mechanical approach is described in detail in ref. [1]. Here, we provide the main points of the theory. The approach describes the HOH generation process using a light field in a coherent state. The description is based on a quantized-Volkov light-electron wave function resulting from a closed-form solution of the time-dependent-Schrodinger equation (TDSE) which in the high photon number limit reads: $\Psi(q,p,t) \propto \psi_0(p)\exp\left(a(t)q^2 + b(t)p^2 + d(t)pq + f(t)p + g(t)q\right)$ (where p and q are the electron momentum and in-phase quadrature of the field [2, 3], respectively and the analytical expressions of $a(t), b(t), d(t), f(t), g(t)$ are given in terms of the parameters of TDSE). The wave function $\Psi(q,p,t)$, which provides the full quantum-mechanical description of the electron-light interaction, includes an arbitrary initial e-wave function $\psi_0(p)$, an arbitrary initial photon number N_0 and the phase ϑ of the driving IR field. This wave function is utilized in order to obtain the back-action of the harmonic generation process in the coherent state of the driving IR field. This back-action appears in the probability distribution of the IR photons after the interaction with the gas phase medium.

When the coherent light state interacts (in single recollision) coherently with n_a atoms towards the generation of XUV radiation (we assume that the XUV emission is the only process which takes place during the recollision, as has been assumed in the semi-classical analysis of Lewenstein *et al.*, ref. [4]), the probability distribution becomes time dependent, since $\Psi(p, q, t)$ is changing at each moment of time within the cycle of the laser field due the interaction with the ionized electron. In this case it can be found that the probability distribution reads $P_n = |A_n(t)\exp\left(i\Phi(t,t_\mathrm{i},t_\mathrm{r})\right)|^2$ where $A_n \propto \exp\left[-(n-(N_0-N_q^{(\mathrm{IR})}))^2/(N_0-N_q^{(\mathrm{IR})})\right]$, N_0 is the photon number of the driving laser field, $Q = \hbar\omega_{\mathrm{XUV}}/\,\hbar\omega_{\mathrm{IR}}$, $N_q^{(\mathrm{IR})} = n_\mathrm{a}Q$ reflects the number of

IR photons absorbed by the n_a atoms of the gas towards XUV emission, $\Phi \approx -\frac{(t-t_r)p_Q(t)^2}{2} + \frac{p_Q(t)A_0}{\omega_{IR}}\sin(\vartheta_i)[1-\cos(\omega_{IR}(t-t_r)] - \frac{p_Q(t)A_0 n}{N_0\omega_{IR}}\cos(\vartheta_i)\sin(\omega_{IR}(t-t_r))$, p_Q(t) is the electron momentum, t_r is the recombination time, ϑ_i is the phase of the IR field at the moment of ionization (t_i), ω_{IR} is the frequency of the IR field and A_0 is the amplitude of the IR field. The parameters p_Q(t), t_r, t_i were obtained using the 3-step semi-classical model [4]. Note, that n_a is a parameter which can be obtained by the detected photon number taking into account the propagation effects and phase matching conditions [5, 6]. For multiple recollisions (interaction with multi-cycle driving laser field) the IR probability distribution can be calculated by coherently summing over the cycles of the driving laser field. This results in a modulated probability distribution with well-confined peak structure (Fig. 1c of the main text of the manuscript), which is directly linked to the HOH spectrum recorded by a conventional XUV spectrometer.

In other words, assuming, that the XUV emission is the only process which takes place during the recollision, a single-recollision-single-atom interaction leads to reduction of the photon number of the initial IR coherent state by a factor of Q ($\hbar\omega_{XUV}/\ \hbar\omega_{IR} = Q$). In a repeating measurement (multi-shot experiment) the probability distribution of the absorbed IR photon number will be continuum which ranges from 0 to Q_{max} IR photons (where Q_{max} is the maximum photon energy of the generated XUV photon). This IR probability distribution is directly associated with the conventional continuum XUV spectrum. In the more realistic case where n_a (non-interacting with each other) atoms coherently interact with the single-cycle IR field the absorbed IR photon number will be n_a x Q. This can be obtained from the TDSE taking into account n_a atomic cores, n_a active electrons and the total IR-laser radiation. For the conditions used in a HHG experiment the atom-atom interaction can be neglected, and the initial TDSE can

be decoupled into independent TDSE's, each for one atom + IR-radiation, the total wave-function being a product of the wave-functions of the individual atoms + IR-radiation. Thus, apart from any propagation effects, each atom contributes equally and independently to the corrections of the total IR field, which in terms of absorbed IR photon number translates to n_{a} x Q. In this case the continuum IR probability distribution, like the continuum XUV spectrum recorded by a conventional XUV spectrometer, can be obtained in a single shot measurement due to the large number of atoms (n_{a}) participating the process.

When n_{a} atoms interact with a multi-cycle IR laser field, each atom can emit a single XUV photon at different cycles of the laser field. In this case the probability distribution of the IR missing photons results from the coherent sum of the states of the IR photons absorbed by the XUV emitters at each cycle of the laser field. The periodicity of the process results in a probability distribution which consists a series of well confined peaks (named IR-harmonic peaks) directly associated with the well known HOH spectra recorded using conventional XUV spectrometers. The peaks in the probability distribution will be at positions n_{a} x q (q is the harmonic order), while the inter-peak spacing will be 2 x n_{a} (the number 2 is resulted by the IR photon number difference between two consecutive harmonics).

In a real HOH generation experiment these distributions are imprinted in the photon number of the outgoing from the medium IR field as $N_0' = N_0 - N_{\mathrm{abs}}^{(\mathrm{IR})} - N_q^{(\mathrm{IR})}$ (where N_0 is the photon number of the non-interacting laser field and $N_{abs}^{(IR)}$ is the number of the absorbed IR photons that do not lead to XUV emission). Since the signal of interest is superimposed on a large background $N_0 - N_{\mathrm{abs}}^{(\mathrm{IR})}$, the XUV/IR correlation approach presented in the main text of the manuscript has been implemented in order to subtract $N_0 - N_{\mathrm{abs}}^{(\mathrm{IR})}$ form N_0' and thus measure the $N_q^{(\mathrm{IR})}$. Additionally, we note that although an accurate calculation of the probability distribution

requires the consideration of the IR laser bandwidth and the propagation effects in the medium, the fundamental properties of the interaction can be adequately explored with the single-color single-atom interaction, as has been done for the calculation of the XUV spectrum in the work of ref. [4]. We note that spatial intensity distribution and propagation effects in the harmonic generation medium can cause subtle dependence of n_a on q. Although such effects can influence the width and spacing of the IR-harmonic peaks, they have not been taken into account in the present work as they were not observable due to the finite resolution of the measurements.

Supplementary Note 2 | Obtaining the position of $N_{q=0}^{(\mathrm{IR})}$ in the IR distribution

The value of the measured photon number n (shown in Fig. 3c of the main manuscript) corresponding to $N_{q=0}^{(\mathrm{IR})}$, the missing number of photons at q=0, is obtained as follows. (1) In Supplementary Fig. 1a we plot the dependence on q of the photon number n measured at the peaks of the distribution, called n_q (black squares). Extrapolating this dependence to q=0 we find $n_0 \approx 2.1 \times 10^8$ photons/pulse (yellow-filled circle). This number defines $N_0 - N_{\mathrm{abs}}^{(\mathrm{IR})}$, the remained IR photon number resulting from the absorption due to processes other than harmonic emission. (2) In Supplementary Fig. 1b we plot the linear dependence of $N_q^{(\mathrm{IR})}$ on q, where $N_q^{(\mathrm{IR})}$ is the number of absorbed (missing) IR photons due to harmonic emission. This is obtained by subtracting n_q from n_0. It is obvious that $N_q^{(\mathrm{IR})}$ is increasing with q and $N_{q=0}^{(\mathrm{IR})} = 0$. In the same way we have obtained the positions of $N_{q=0}^{(\mathrm{IR})}$ in the IR distributions shown in Figs' 3d and 4 of the main text of the manuscript.

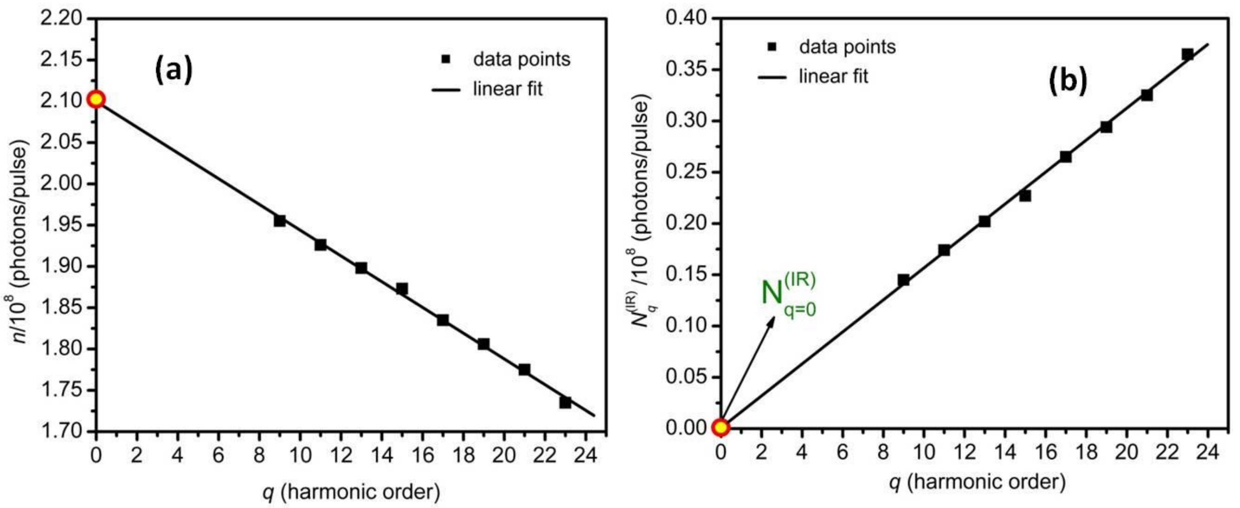

Supplementary Figure 1|Linear dependence of the IR photon number on q. (**a**) Dependence of the measured photon number n of the peaks of the distribution (black squares) on q. The yellow-filled circle shows the value of $n_0 \approx 2.1\text{x}10^8$ photons/pulse which has been found by extrapolating the linear dependence to q=0. (**b**) Linear dependence of $N_q^{(\mathrm{IR})}$ on q. The yellow-filled circle shows the value of $N_{q=0}^{(IR)} = 0$.

Supplementary Note 3|XUV energy distribution

Although the understanding of the distribution of the XUV energy requires further theoretical analysis for obtaining the wave function of the XUV radiation, for the sake of completeness we provide the probability distribution of the PMT signal. A broad XUV distribution appears around the mean value of XUV photons number. After applying the approach described in the main text of the manuscript, the XUV distribution reveals the peak structure shown in Supplementary Fig. 2. This distribution is consistent with the corresponding IR distribution, as it exhibits similar harmonic peak structure having a peak spacing corresponding to consecutive harmonics. Note, however, that due to the rough measurement of the XUV photon number and the balance with the IR signal which takes place in data analysis procedure, the x-axis cannot be used for the

accurate calibration of the measured photons. The assignment of the harmonic orders on the XUV-harmonic distribution peaks and the grey shaded areas are based on the calibration of the IR distribution shown in Fig. 3c and 3d of the main text of the manuscript.

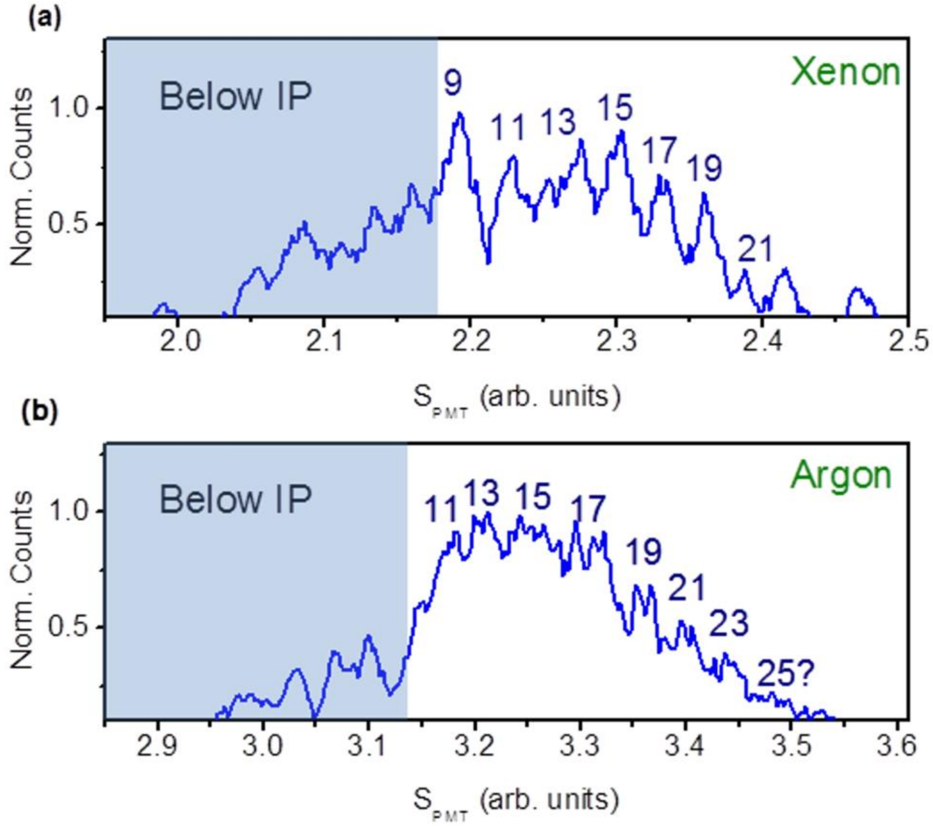

Supplementary Figure 2│Energy distribution of the XUV radiation recorded by the PMT. (**a**) XUV harmonic peak structure which corresponds to Fig.3c of the main text of the manuscript. (**b**) XUV harmonic peak structure which corresponds to Fig.3d of the main text of the manuscript. Note, that in contrast with IR-harmonic distribution (Fig. 3c and 3d), in these graphs the higher harmonics correspond to higher values of S_{PMT}, as is expected.

Supplementary references

1. Gonoskov, I. A., Tsatrafyllis, N., Kominis, I. K., and Tzallas, P., Quantum optical signatures in strong-field laser physics: Infrared photon counting in high-order-harmonic generation. *Sci. Rep.* **6**, 32821; doi:10.1038/srep32821 (2016).

2. Mandel, L. and Wolf, E. *Optical Coherence and Quantum Optics* (Cambridge Univ. Press, 1995).

3. Schleich, W. P. *Quantum Optics in Phase Space* 1st edn (John Wiley & Sons, 2001).

4. Lewenstein, M., Balcou, Ph., Ivanov, M. Yu., L'Huillier, A., Corkum, P., Theory of high-harmonic generation by low frequency laser fields. *Phys. Rev. A* **49**, 2117-2132 (1994).

5. Eberly, J. H., and Fedorov, M. V., Spectrum of light scattered coherently or incoherently by collection of atoms. *Phys. Rev. A* **45**, 4706-4712 (1992).

6. Constant, E., Garzella, D., Breger, P., Mevel, E., Dorrer, E., Le Blanc, C., Salin, F., and Agostini, P., Optimizing High Harmonic Generation in Absorbing Gases: Model and Experiment. *Phys. Rev. Lett.* **82**, 1688-1671 (1999).
